# Strengthening health system to improve immunization for migrants in China

**DOI:** 10.1186/s12939-016-0504-8

**Published:** 2017-07-01

**Authors:** Hai Fang, Li Yang, Huyang Zhang, Chenyang Li, Liankui Wen, Li Sun, Kara Hanson, Qingyue Meng

**Affiliations:** 1 China Center for Health Development Studies, Peking University, Beijing, China; 2 Department of Health Policy and Management, School of Public Health, Peking University, Beijing, China; 3 London School of Hygiene and Tropical Medicine, London, UK

**Keywords:** Immunization, Vaccine, EPI, Migrant, China

## Abstract

**Background:**

Immunization is the most cost-effective method to prevent and control vaccine-preventable diseases. Migrant population in China has been rising rapidly, and their immunization status is poor. China has tried various strategies to strengthen its health system, which has significantly improved immunization for migrants.

**Methods:**

This study applied a qualitative retrospective review method aiming to collect, analyze and synthesize health system strengthening experiences and practices about improving immunizations for migrants in China. A conceptual framework of Theory of Change was used to extract the searched literatures. 11 searched literatures and 4 national laws and policies related to immunizations for migrant children were carefully studied.

**Results:**

China mainly employed 3 health system strengthening strategies to significantly improve immunization for migrant population: stop charging immunization fees or immunization insurance, manage immunization certificates well, and pay extra attentions on immunization for special children including migrant children. These health system strengthening strategies were very effective, and searched literatures show that up-to-date and age-appropriate immunization rates were significantly improved for migrant children.

**Conclusions:**

Economic development led to higher migrant population in China, but immunization for migrants, particularly migrant children, were poor. Fortunately various health system strengthening strategies were employed to improve immunization for migrants in China and they were rather successful. The experiences and lessons of immunization for migrant population in China might be helpful for other developing countries with a large number of migrant population.

## Background

Immunization is the most cost-effective method to prevent and control vaccine-preventable diseases [1, 2]. Immunization plays an important role to reduce mortalities for children and protect adult health at the same time. It is also an important aspect of public health, which needs efficient immunization programs and specific implementation strategies. China has been the world’s most populous country. Before the 1950s, morbidities of infectious diseases were very high and hurt people’s health due to limited capabilities of prevention and control, and more than 10 million cases of measles, polio, diphtheria and pertussis occurred each year [3]. After the founding of the People’s Republic of China, China eliminated small pox and polio and substantially decreased morbidities of tuberculosis (TB), diphtheria, pertussis, tetanus, and measles through an Expanded Program on Immunization (EPI) [3]. Before 2007, 5 basic vaccines including Bacille Calmette Guerin (BCG), polio, diphtheria tetanus pertussis (DTP), measles, and hepatitis B were offered to children, and the EPI program expanded to 14 vaccines in 2007 including BCG, polio, DTP, measles, hepatitis B, Td (tetanus and diphtheria), hepatitis A, Japanese encephalitis, A + C meningococcal polysaccharide, mumps, rubella, hemorrhagic fever, anthrax, and leptospirosis [4].

After the reform and opening up policy in 1978, internal migrant population in China has been rising rapidly. Migrants in China refer to people who were not living in their household registration (Hukou) places (not the same counties or cities of Hukou registration) and migrants had to be back to their Hukou places in the future. In 2014, China had about 253 million migrant people [5], and China will have 291 million migrant people by the year of 2020 [6]. It was estimated that approximately 15–23% of children were migrating in the 1990s [7]. Most of these migrant children were less than 24 months, and they had to stay with their parents. The first 24 months are critical to migrant children’s immunization. When they became older, they could stay with their grandparents in hometowns (became left-behind children). 2010 China’s Census showed that there were 35.81 million migrant children, and 13 of 100 Chinese children were migrating [8]. In 2014, 60% of people migrated with spouse and children [6].

There are mainly four reasons for the rising migrant population in China. First, economic development led to more movements of people and goods, and domestic markets became larger. Second, inequality of economic development between eastern China and central/western China attracted a large number of people to move to eastern areas for jobs and business. Third, China’s household registration system (Hukou) did not allow people to move permanently, and most migrations were temporary. Fourth, a certain number of people moved to other places because they violated the local family planning policy and were not able to live in their hometowns any more. Migrant adults often brought young children with them or had newborn children delivered while they were migrating. Migrant adults violating the family planning policies definitely had to bring children with them.

The rising migrant population (particularly children) brought serious issues to immunization in China. The local immunization authorities did not know the real number of children who needed immunization, as migrants often did not report their migrant activities to authorities of input and output areas. Particularly migrant children did not need to report to the local living authorizes while they migrated with their parents. There was a significant gap between local resident children and migrant children for immunization up-to-date and age-appropriate rates [9]. Outbreaks of infectious diseases such as measles cases in Wuhan in 1995 and in Beijing in 2000, were all related to migrant children [10, 11].

China realized the serious immunization issues for migrants, and various strategies were integrated together in the health system. Vaccines covered in the EPI program were mainly for children, so the immunization issues for migrants were focused on migrant children. Immunization up-to-date and age-appropriate rates for migrant children were improved, but it was still lower than the local resident children in some places. It will be great to summarize experiences and lessons from China’s immunization for migrant children, which will provide suggestions and recommendations to other developing countries with a large number of migrant people.

### A brief introduction about China’s immunization policies and practices

There were three important stages of China’s immunization policies and practices. The first stage was from 1950 to 1977. At the beginning of this stage, infectious disease status in China was very serious. During the period of 1950–1965, the measles morbidity rate was 590/10,000 on average, and polio morbidity rates were estimated to be 32–1,500/100,000 [12]. Approximately 10 million Chinese people were infected in the pandemic of measles in 1959, and 300,000 died [12]. After the founding of the People’s Republic of China, immunization was rapidly carried out. During the first eight months of 1950, 8% of Chinese population (approximately 40 million) was immunized with small pox vaccines. At the end of 1952, 500 million people (almost the entire population in China) were immunized with small pox vaccines, and small pox was eliminated in China in the early 1960s.

The second stage was from 1978 to 2005. In 1978, China responded to the EPI recommendations by the World Health Organization (WHO), and planned to provide all Chinese citizens 4 types of vaccines: BCG, polio, DTP, and measles to prevent and control 6 infectious diseases: TB, poliomyelitis, diphtheria, pertussis, tetanus, and measles. In terms of immunization funding, the Central Government provided vaccines and immunization working funds to less developed provinces, and other provinces were responsible for their own funds. Vaccines were provided to individuals without charges, but people had to pay some immunization services fees as incentives to immunization staffs and organizations or people had to buy immunization insurance. Immunization insurance was used for compensation payment, if one child was immunized but still infected. It often covered up to 7 years old, and the insurance premium was charged by year. Charging individuals’ immunization services fees or insurance negatively affected immunization, and was stopped in 2005.

Even if China made significant achievements on immunization, the immunization issues for migrant children in China were mainly in the second stage. The immunization up-to-date and age-appropriate rates for migrant children were often less than 50%, which were far lower than the 85% national target rate. For example, none of 17 migrant children got polio immunization in a farming firm in Shenzhen, and 5 of them were infected with polio in 1986 [13]. In Ninghai County, Ningbo City, Zhejiang Province, only 29.26% of migrant children (a total of 1097) had immunization certificates, and only 7.93% had all 4 vaccines of BCG, DTP, polio, and measles immunized up to one year old [14]. During the period of 1985–1991 in Shenzhen City, Guangdong Province, there were 16 children infected with polio, and 13 (81.2%) were migrant children [15]. In 1991–1992, only 32.9% of children migrated from other cities to Wuhan City, Hubei Province were immunized with all 4 vaccines: BCG, DTP, polio, and measles, and the immunization rates were only 60% for children migrated within Wuhan City and 100% for local Wuhan children without migrant experiences [16]. 40% of children did not complete 3 doses of polio immunization within 6 months after delivery in Qingyang County, Anhui Province in 1991, and all of these children migrated with their parents to other places [17].

The third stage is from 2005 to present. In 2005, the State Council announced “Regulation about Vaccine and Immunization Management” and stopped charging immunization service fees or immunization insurance for 5 basic EPI vaccines: BCG, DTP, polio, measles, and hepatitis B. Immunization certificates were emphasized again for migrant population, and migrants were treated same as local residents according to this policy. As China had more financial resources and realized the importance of immunization, the Chinese Government quickly expanded the EPI program. In 2007, “Implementation Plan of Expanding National EPI Program” was announced, which increased types of vaccines, strengthened surveillance of EPI quality, and improved immunization effectiveness [4]. Fourteen vaccines (see above) were provided to individuals free of charge against 15 diseases. Immunization rates of newly added vaccines into the EPI program were significantly improved. Reported immunization rates for children at the township level were more than 90% for 5 basic vaccines included in the previous EPI program, and reported immunization rates for newly added vaccines were more than 70%.

During the third stage, immunization issues for the migrant children were reduced substantially due to the health system reform, revised immunization policies, and economic development. For examples, in 2012, 96.68% of 602 migrant children in Jiangdu City, Henan Province had age-appropriate 5 basic vaccines immunized [18]. In Qidong City, Jiangsu Province, 96.93% of migrant children had immunization certificates in 2012, but the age-appropriate 5 basic vaccines’ immunization rate was only 79.75% and still significantly lower than local resident children (98.75%) [19].

## Methods

This study applied a qualitative retrospective review method aiming to collect, analyze and synthesize health system strengthening experiences and practices about improving immunizations for migrants in China.

### Search terms and strategy

We searched databases of PubMed, China National Knowledge Infrastructure (CNKI), and Wan-Fang for migrant immunizations in China, and the key words included “EPI”; “Expanded Program on Immunization”; “Immunization”; “Migrant”; “Migration”; and “China”. The search did not restrict publication dates, as we would like to include all the relevant information. Government policies and laws about immunization were also reviewed, as they were often relevant to immunizations for people with low socioeconomic status including migrants. Published articles often examined the effectiveness of these government policies and laws in terms of immunization rates and morbidities of infectious diseases.

PubMed is the largest English publication database, and should cover all the English publications about migrant immunization in China. China National Knowledge Infrastructure (CNKI) and Wan-Fang are two largest databases for Chinese literatures. The concept of National Knowledge Infrastructure (NKI) was introduced by the World Bank in 1998, and China NKI was developed by Tsinghua University with supports from Chinese government ministries, academia, publishers, etc. Wanfang database was a leading information contents provider in China, and had been a task force of the Institute of Scientific & Technological Information of China (ISTIC), China Ministry of Science & Technology, since 1950s. Because most of immunizations studies for migrants were published in Chinese, these two Chinese databases were also searched besides PubMed database.

### Inclusion and exclusion criteria

We aimed to include all studies analysing immunizations for migrants. In order to obtain sufficient information relevant to the study objectives, we included all articles describing or analysing the detailed process of designing and implementing general immunization policies and processes related to migrants, and then extracted information related to health system strengthening. The judgement on relevant to health system strengthening was based on a conceptual framework of Theory of Change including four parts: content, process, effect, and context.

Given that the study did not seek to quantify outcomes, but to ensure that all relevant information relevant to immunizations for migrants were included, even if implicitly, the study included peer-reviewed papers but also policy documents, commentaries, viewpoints, project reports and policy documents. There were no restrictions on the study designs and methods. We did not conduct conventional methodology quality appraisal including risk of bias of the studies included in this review, however the publications were appraised in terms of significance, level of detail and relevance to the research topics.

Two researchers independently screened the abstracts and titles and discussed with the lead author to achieve consensus. The lead author screened all the full texts and other co-authors checked all the full texts in order to ensure no important documents were missed.

### Data extraction and conceptual framework

A conceptual framework of Theory of Change was used to extract the searched literatures and examine how China strengthened health system to improve immunization for migrants [20]. Theory of Change includes 4 parts: content, process, effect, and context. First, we studied the contents of immunization policies and health system strengthening strategies related to migrant population in China. Second, we analyzed processes of these policies and strategies, particularly the implementation process. Third, we examined the effects of policies and strategies by linking immunization rates and morbidities with migrant population from the literatures and published statistics. Finally, we investigated what context factors could explain the experiences and lessons of immunization for migrant population.

Each of these 4 parts and characteristics of immunization were then operationalized to specific questions by a multi-disciplinary international expert group collaborating on a larger project to synthesise the experience of China in health system development and lessons for other countries undergoing similar developments. The development was through an initial face to face workshop followed by virtual interaction to refine the framework after the initial stages of analysis.

We analysed and synthesised the exacted qualitative and textual data using a framework synthesis approach [21]. The rationale of this synthesis method is that for the large amounts of textual data extracted from primary studies, framework synthesis offers a highly structured approach to organising and analysing data. Thus immunization issues for migrants were categorized and matched onto each domain of the conceptual framework, while allowing for new attributes to emerge. The attributes were then synthesised and reorganised hierarchically, for example to identify related and divergent attributes.

As a supplementary qualitative approach, we also interviewed 2 government officials and 2 health experts including: one official in China CDC, one official in the Disease Control Bureau of National Health and Family Planning Commission, and two experts from medical associations. Laws and government policies related to immunization and migrant population were also reviewed and analyzed. The information from these official and expert interviews, laws, and government policies as a qualitative approach was very helpful and supplementary to the literature reviews within the research framework of the Theory of Change.

## Results

### Search results

**Fig. 1:**
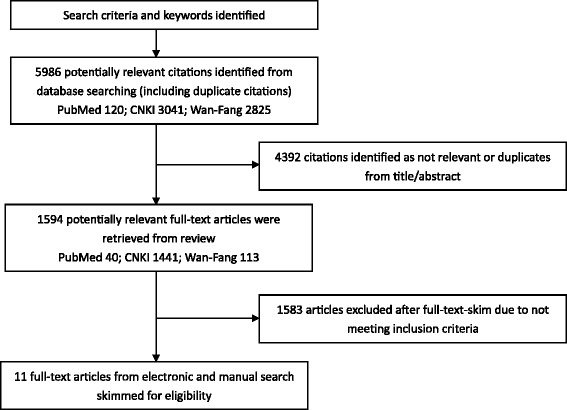
Search and Screening Flow Chart

Figure 1 shows a search and screening flow chart. After search criteria and keywords were identified, a total of 5986 potentially relevant articles (including duplicate articles) were searched from 3 databases: PubMed (120 articles), CNKI (3041 articles), and Wan-Fang (2825 articles). 4392 articles were dropped based on duplicates, and title/abstract screenings. 1594 potential relevant full-text articles were retrieved for review from 40 articles from PubMed, 1441 articles from CNKI, and 113 articles from Wan-Fang. After full-text skim, 1583 articles were excluded because they did not study immunization or immunization for migrant population in China, and 11 full-text articles [22–32] were left for careful review and their citations were reported in the reference list.

### Description of included studies

Four national laws and government policies [33–36] were relevant to immunizations for migrants in China, and they were also examined carefully. Based on the literature reviews, government policies and laws about immunization and migrant population, and expert/official interviews, the present study summarized three successful health system strengthening strategies to improve immunization for migrant population in China.

### Three successful Strategies to improve immunization for migrant population

Table 1 shows a summary of 11 searched literatures and 4 national laws and policies related to migrant immunization. Based on the research framework of Theory of Change, Table 2 shows three 3 successful strategies in terms of content, process, effect, and context.

**Table 1:** A summary of searched literatures and national polices and laws

Ref #	Authors	Year	Study Sample	Study Location	Main Findings
22	You	1992	All the children up to 7 years old	Rugao City, Jiangsu Province	A newborn baby had to pay for a lump-sum immunization insurance for 7 years (18–25 Yuan), right after the baby was discharged from maternal and children hospitals. The lump-sum immunization insurance up to 7 years was a barrier for migrant children to receive immunizations.
23	Xu et al.	1995	6121 migrant children less than one year old	Linyi City, Shandong Province	A shorter period of immunization insurance (only one year after the delivery instead of 7 years) used for migrant population with much lower premium and it was very effective for migrant children to receive 5 basic vaccines in the first year.
24	Li et al.	2008	2859 migrant children aged 0–7	Baicheng City, Jilin Province	The rate of appropriate immunizations for migrant children increased from 80% in 2004 to 95% in 2006, after this national policy "Regulation about Vaccine and Immunization Management" was implemented in 2005
25	Liao	2008	1859 migrant families	Chengdu City, Sichuan Province	A positive relationship between immunization certificates and age-appropriate immunization rates was found. 95% of migrant children in Chengdu City, Sichuan Province had immunization certificates and their age-appropriate immunization rates were more than 90%.
26	Gao	2008	120 migrant children	Yangzhou City, Jiangsu Province	A positive relationship between immunization certificates and age-appropriate immunization rates was found. 95.8% of migrant children had immunization certificates in Yangzhou City, Jiangsu Province, and their 5 vaccine’immunizations rates were 87.5%
27	Yin et al.	2008	436 migrant children	Panzhihua City, Sichuang Province	A positive relationship between immunization certificates and age-appropriate immunization rates was found. 97.71% of migrant children in Panzhihua City, Sichuan Province had immunization certificates and 5 vaccines (except hepatitis B) were more than 85%
28	Nakano et al.	1997	91 children	Myanmar border, Yunan Province	The former family planning policy had been believed to be one of most important reasons for the low immunization rates for migrant children
29	Li et al.	1994	194 migrant children	Wujiang City, Jiangsu Province	Migrant families had more children violating the family planning policy. 13.92% of 194 migrant children were out of family planning policies in Wujiang City, Jiangsu Province in 1993.
30	Wang	1996	61 polio cases	Wuhua County, Guangdong Province	Investigated 61 polio outbreak cases in Wuhua County, Guangdong Province in 1993, and found that 39 cases were for children violating the family planning policy or migrant children and none of them had a complete polio immunization history
31	Han et al.	2014	1610 migrant children	Guangdong Province	Migrant children living in a poor family had a low up-to-date immunization rates.
32	Zhang and Wang	1999	All migrant children	Jiangle County, Fujiang Province	Examined immunization for special population in Jiangle County, Fujian Province, and found this policy "Management Plan for Special Population’s Expanded Program for Immunization" was very effective to improve immunization for migrant children.
33	National People's Congress	1989	Entire population	China	People’s Republic of China’s Law about Preventing and Controlling Communicable Diseases (national law)
34	National People's Congress	2004	Entire population	China	Revision of People’s Republic of China’s Law about Preventing and Controlling Communicable Diseases (national law)
35	State Council	2005	Entire population	China	Regulation about Vaccine and Immunization Management (national policy)
36	Ministry of Health	1998	Entire population	China	Management Plan for Special Population’s Expanded Program for Immunization (national policy)

**Table 2:** Three successful strategies to improve immunization for migrant population by Theory of Change

Content	Process	Effect	Context
Stop charging immunization service fee or immunization insurance.	Immunizations for migrant population were responsible by the local living places, while they were not in their original Hukou places. After 2005, all the immunization funds were allocated directly to the local immunization clinics based on the total population in each area, and migrants were also included. Immunization clinics had financial incentives to provide immunization services to migrants.	In 2004, only 80% of migrant children in Baicheng City, Jilin Province had appropriate immunizations, and this rate increased to more than 95% in 2006 right after “Regulation about Vaccine and Immunization Management” was implemented in 2005 [24].	The revision of “People’s Republic of China’s Law about Preventing and Controlling Communicable Diseases” in 2004 specified that EPI immunizations should be free of charge. In 2005, the State Council announced “Regulation about Vaccine and Immunization Management” and specified that five EPI immunizations should be provided to people free of charge without extra immunization service fees or immunization insurance.
Manage immunization certificates well.	Immunization certificates were checked when children received annual physical examinations and got into kindergartens and schools. Catch-up programs were provided to children without up-to-date immunization records in their immunization certificates or without immunization certificates.	In 2007, 95% of migrant children in Chengdu City, Sichuan Province had immunization certificates and their age-appropriate immunization rates were more than 90% [25]. In 2008, 95.8% of migrant children had immunization certificates in Yangzhou City, Jiangsu Province, and their 5 vaccine’ immunizations rates were 87.5% [26].	“Regulation about Vaccine and Immunization Management” in 2005 specified that the immunization certificate should be issued to newborn babies within one month after delivery by the local residential immunization stations.
Pay more attentions to special children for their immunization, including migrant children, children violating family planning policies, and children without ability to pay immunization service fees or insurance.	For children without ability to pay, immunization service fees or insurance were waivered. Local immunization staffs tried to search for special children without up-to-date immunizations, and provide appropriate immunization services to them. The local immunization staffs tried to collect information about newborn children by collaborating with other departments. It was allowable not to record their real names and/or addresses for children violating family planning policies, but the immunization certificates were still issued to them.	After the immunization policy for special population was implemented in Jiangle County, Fujian Province, 80% of migrant people received timely immunization information and 90% of parents with migrant children went to immunization clinics to receive EPI immunizations [32]. Catch-up immunizations were provided to children without up-to-date immunizations, and immunization insurance was also waived.	In 1998, the former Ministry of Health announced “Management Plan for Special Population’s Expanded Program for Immunization” and tried to improve immunization work for special children.

#### Strategy 1: Stop charging immunization service fee or immunization insurance

In 1989, China announced “People’s Republic of China’s Law about Preventing and Controlling Communicable Diseases”, but this law did not specify whether children should be charged for vaccines and/or immunization service fees [33]. Immunization insurance was widely used as the methods to charge the immunization service fees in China. You (1992) examined immunization insurance in Rugao City, Jiangsu Province and found a newborn baby had to pay for a lump-sum immunization insurance for 7 years (18–25 Yuan), right after the baby was discharged from maternal and children hospitals [22]. A significant portion of immunization insurance premium were used for immunization staff incentives and cold-chain system [22], which should actually have been taken care by governments and/or health departments. Xu et al. (1995) studied a shorter period of immunization insurance (only one year after the delivery instead of 7 years) used for migrant population with much lower premium in Linyi City, Shandong Province, and found it was very effective for migrant children to receive 5 basic vaccines in the first year [23].

The revision of “People’s Republic of China’s Law about Preventing and Controlling Communicable Diseases” in 2004 specified again that immunizations should be free of charge for EPI vaccines [34]. In 2005, the State Council announced “Regulation about Vaccine and Immunization Management” [35] and specified that immunization for 5 type 1 vaccines: BCG, DTP, hepatitis B, polio, and measles were provided to people free of charge without extra immunization service fees. Above two national laws or policies were mainly contexts for this successful strategy. This regulation by the State Council required the following process: all the residents were included regardless of their Hukou, and immunization for migrant population was responsible by the living places if different from Hukou places. The searched literatures showed this strategy was very effective. Li et al. (2008) analyzed expanded program on immunization for migrant children in Baicheng City, Jilin Province, and found that “Regulation about Vaccine and Immunization Management” successfully improved immunization rates [24]. They found that the rate of appropriate immunizations for 2016 migrant children increased from 80% in 2004 to 95% in 2006, after this regulation was implemented in 2005 [24].

#### Strategy 2: Manage immunization certificates well

According to EPI polices in China, an immunization certificate was issued to children, and all the immunization history were recorded on this certificate. This immunization certificate was kept by parents. At the same time, an immunization card with the same information was set up and kept by the immunization clinics. Strategy 2’s content was immunization certificates. “Regulation about Vaccine and Immunization Management” in 2005 as the main context specified the following process: 1) the immunization certificate needed to be issued to newborn babies within one month after delivery by the local residential immunization clinics; 2) during the period in which children were not living in their original places (for migration), the current living places instead of original Hukou places were responsible for their immunization; 3) immunization certificates were checked when children received annual physical examinations and got into kindergartens and schools; and catch-up programs were provided to children without up-to-date immunization records in their immunization certificates or without immunization certificates [35]. Several studies found immunization certificates significantly improved age-appropriate immunization rates. For example, in 2007, 95% of migrant children in Chengdu City, Sichuan Province had immunization certificates and their age-appropriate immunization rates were more than 90% [25]. In 2008, 95.8% of migrant children had immunization certificates in Yangzhou City, Jiangsu Province, and their 5 vaccine’ immunizations rates were 87.5% [26]. 97.71% of migrant children in Panzhihua City, Sichuan Province had immunization certificates and 5 vaccines (except hepatitis B) were more than 85% [27].

#### Strategy 3: Pay more attentions on special children for their immunization

Special children were the main content of Strategy 3. Nakano et al. (1997) examined migrant population bypassed by polio vaccination programs in Yunnan Province, and found that the former family planning policy was one of most important reasons for the low immunization rates for migrant children [28]. This was because the major reason for them to migrate from their hometowns to other places was violating the formal family planning policy (so-called one child policy) [28]. For example, 13.92% of 194 migrant children were out of family planning policies in Wujiang City, Jiangsu Province in 1993 [29]. Wang (1996) investigated 61 polio outbreak cases in Wuhua County, Guangdong Province in 1993, and found that 39 cases were for children violating the family planning policy or migrant children and none of them had a complete polio immunization history [30]. Socioeconomic status of migrant children’ parents was relatively low in terms of education, occupation, living conditions, and incomes. The primary caregiver’s occupations for migrant children in Guangdong Province in 2010 (commercial staff, professional, private owner, and housewife) significantly increased the odds of their children being up-to-date for immunizations compared with workers; family income more than 3000 Yuan/person/year increased child’s odds ratio of being up-to-date; children living in purchased houses were more likely to receive up-to-date immunization than children living in renting houses [31].

Children violating the family planning policy or living in the poor family with low socioeconomic parents were regarded as special population. In 1998, the former Ministry of Health announced “Management Plan for Special Population’s Expanded Program for Immunization” [36] as the main context of Strategy 3, and special population mainly included migrant children, children violating the family planning policy, and children living in the ethnic minority, remote and/or poor areas. This policy had the following 7 processes: 1) all the children had equal rights to receive EPI immunizations regardless of local resident children and migrant children, children allowed by the family planning policy and children violating the family planning policy, children purchased and not purchased the immunization insurance, and children with and without abilities to pay for the immunization service fees; 2) for those poor people, immunization service fees were waivered; 3) for each 3 months in the urban areas and each 6 months in the rural areas, local immunization staffs searched for special children without up-to-date immunizations, and provided appropriate immunization services to them. Immunization staffs tried to reach migrant children in their parents’ working sites or rented houses at off-working hours. The local immunization staffs tried to collect information about newborn children by collaborating with departments of public security, family planning, maternal and child health, women’s federation etc.; 4) it was allowable not to record their real names and/or addresses, but the immunization certificates should still be issued to special children; 5) special children were told for dates and locations of next immunizations; 6) for those children without immunization certificates, all the vaccines were immunized again within one year since moving into the local areas; and 7) the immunization fund were allocated according to the combined number of local resident children and migrant children, and more management fund, immunization staffs, and vaccines were allocated to migrant children than local resident children by the local governments. Zhang and Wang (1999) examined immunization for special population in Jiangle County, Fujian Province, and found this policy was very effective [32]. After the immunization policy for special population was implemented in Jiangle County, Fujian Province, 80% of migrant people received timely immunization information and 90% of parents with migrant children went to immunization clinics to receive EPI immunizations [32].

## Discussion

Immunization for migrants in China is a systematic issue, and a single strategy is not able to address it successfully. Health system strengthening strategies in China have been employed together. As long as China still employs the Hukou resident registration, there will be more and more migrants due to the rapid economic development. The former family planning policy will not be an important issue for migrants’ immunization any more.

The present study found that 3 strategies were mainly effective to improve immunization for migrants in China, which were important parts of a complete immunization policy including immunization coverage, funding allocations, and performance evaluations. For example, in 1998 the former Ministry of Health announced “Management Plan for Special Population’s Expanded Program for Immunization” [36], and migrants were regarded as an important part of special population. Therefore, immunizations were provided to migrant children, even if their parents were not able to pay for immunization service fees or immunization insurance. However, some local governments did not have a sustainable strategy to finance immunization for migrants and immunization clinics did not have strong financial incentives to carry out immunization for migrants. The national policy required local governments to pay more attentions to immunizations for special population, but the implementation levels in different provinces were diverse. Another example was the immunization certificates. Each child was required to have its own immunization certificate to record immunization history, but the rates of migrant children having immunization certificates were still very low before “Regulation about Vaccine and Immunization Management” was announced by the State Council in 2005.

In order to strengthen the entire health system, especially immunization in the public health sector, a national policy about financing EPI program was provided. The most important part of this EPI program was how to allocate the funds. Based on China’s experiences, the immunization funds including migrants had been provided by the Central Government. China started to provide immunization funds to each province according to the total population including migrants in 2005 according to “Regulation about Vaccine and Immunization Management” and stopped charging immunization service fees or immunization insurance. In 2007, China expanded EPI program from 5 vaccines to 14 vaccines according to “Implementation Plan of Expanding National EPI Program”, and at the same time special immunization funds from the Central Government were allocated to each province to purchase vaccines and cover immunization services fees. In 2009, the essential public health equalization program included immunization, and allocated a proportion of funds to EPI immunization. All these immunization financing strategies were used to all the population including migrants, so migrants had been included into the EPI program completely and the financing strategies ensured that all migrants had the same access to immunization as local residents. In addition, local governments provided extra funds to the immunization clinics for constructions, cold chain system, equipment, and operation expenses.

Immunization for migrants also needed other strategies besides financing mechanism. Immunization certificates were emphasized again in “Regulation about Vaccine and Immunization Management” in 2005. More attentions were paid to migrant children for their immunization according to “Implementation Plan of Expanding National EPI Program” in 2007. The hospital delivery subsides for rural population including migrants provided funds for rural maternal women to deliver babies in hospitals since 2009. In order to reduce newborn mortality rates, immunizations for newborn babies were paid more attentions.

However, maintaining high routine immunization coverage for migrants and hard-to-reach populations was really challenging [37]. For example, even for the nationwide measles supplementary immunization activity in 2010, the immunization rate for migrant children in the capital city: Beijing was only 83.4%, which was significantly lower than the official report rate of 96% among all eligible children in Beijing [38].

## Conclusions

After reform and opening up policy in 1978, there were more and more migrant people in China. Due to the Hukou registration policy, people were not able to move permanently without permission. Charging immunization service fees for vaccines included in the EPI program during the period of 1985–2005 made immunizations for migrant children even more difficult. The family planning policy led more people to migrate to other places for baby deliveries or avoiding punishments, and they did not dare to report newborn babies to the local authorities and get immunizations for them.

A sustainable financing mechanism was the key to immunization work, especially for migrants. Both vaccines and immunization service fees needed to be provided by governments, and people did not pay for immunization covered in EPI program. Migrants were treated same as local residents for immunization. Immunization certificates were issued to all the immunized people and were checked for school enrollments. It was worth to study and review immunization lessons and experiences for migrant children in China, which might be useful for other developing countries with rising migrant population.
